# Cell Culture Assay for Human Noroviruses

**DOI:** 10.3201/eid1307.070131

**Published:** 2007-07

**Authors:** Martin C.W. Chan, Y.P. Wong, Wai K. Leung

**Affiliations:** * The Chinese University of Hong Kong, Hong Kong Special Administrative Region, People’s Republic of China

**Keywords:** human norovirus, in vitro cell culture assay, letter

## To the Editor

We read with great interest the article on human norovirus (hNoV) by Straub et al. (*1*). By using 3-dimensional aggregates of a highly differentiated intestinal epithelial cell line, the investigators claimed to have established an in vitro cell culture model that “support[s] the natural growth of human noroviruses.” While the authors provide compelling evidence of successful virus infection through microscopy, hybridization of viral RNA after 5 passages in cell culture, and preliminary evidence of viral RNA replication through limiting dilution PCR, we question the level of virus replication that is actually achieved in this system.

Straub et al. demonstrate through fluorescent in situ hybridization the presence of viral RNA through 5 passages in his system. This phenomenon could be similar to the findings of Duizer et al. (*2*), if the level of replication simply maintained the viral titer. Therefore, we argue that virus replication curve, estimated by using quantitative real-time PCR or semiquantitative endpoint dilution PCR with the end-dilution of each sample from different time points in this system, will conclusively determine the suitability of this model as a productive virus replication system. To support our hypothesis, we point to the pig model for hNoV infectivity (*3*). In that study investigators failed to observe an increase in viral shedding from symptomatic piglets upon serial passage, despite successful intracellular detection of viral RNA and newly synthesized virus-encoded protein in host cells dying of apoptosis. This suggests that the demonstration of cytopathic effect and virus internalization in cells alone may not provide direct evidence of productive virus replication. In conclusion, although we acknowledge that Straub et al. have provided evidence of successful hNoV infection in vitro, we suggest subsequent studies to characterize the level of virus replication in this system.
