# Supplementary material for: Cell Culture Assay for Human Noroviruses
Source: Emerg Infect Dis. 2007 Jul;13(7):1117–8. doi: 10.3201/eid1307.070131 (PMC2878243; doi:10.3201/eid1307.070131)
Supplement: Related texts

# Reply: In Response

**Authors:** Timothy M. Straub, Kerstin Höner zu Bentrup, Patricia Orosz-Coghlan, Alice Dohnalkova, Brooke K. Mayer, Rachel A. Bartholomew, Catherine O. Valdez, Cynthia J. Bruckner-Lea, Charles P. Gerba, Morteza A. Abbaszadegan, Cheryl A. Nickerson

**Affiliations:** * Pacific Northwest National Laboratory, Richland, Washington, USA; † Tulane University School of Medicine, New Orleans, Louisiana, USA; ‡ University of Arizona, Tucson, Arizona, USA; 1 Current affiliation: Arizona State University, Tempe, Arizona, USA

We appreciate the comments provided by Chan et al., in response to our recently published article (*1*). The specific aim of our project was to develop an in vitro cell culture infectivity assay for human norovirus (hNoV) to enhance risk assessments when these viruses are detected in water supplies. Reverse transcription (RT) qualitative or quantitative PCR are the primary assays for waterborne hNoV monitoring. However, these assays cannot distinguish infectious from noninfectious virions. When hNoV is detected in water supplies, information provided by our infectivity assay will improve risk assessment models and protect human health, regardless of whether we are propagating hNoV. Indeed, in vitro cell culture infectivity assays for the waterborne pathogen *Cryptosporidium parvum* that supplement approved fluorescent microscopy assays do not result in amplification of the environmentally resistant hard-walled oocysts (*2*). However, identification of life cycle stages in cell culture provides evidence of infectious oocysts in a water supply.

Nonetheless, Leung et al.’s assertion regarding the suitability of our method for the in vitro propagation of high titers of hNoV is valid for the medical research community. In this case, well-characterized challenge pools of virus would be useful for developing and testing diagnostics, therapeutics, and vaccines. As further validation of our published findings, we have now optimized RT quantitative PCR to assess the level of viral production in cell culture, where we are finding increases in viral titer. The magnitude and time course of these increases is dependent on both virus strain and multiplicity of infection. We are currently preparing a manuscript that will discuss these findings in greater detail, and the implications this may have for creating viral challenge pools.

## References

[1] Straub TM, Honer zu Bentrup K, Orosz-Coghlan P, Dohnalkova A, Mayer BK, Batholomew RA, In vitro cell culture assay for human noroviruses. Emerg Infect Dis. 2007;13:396–403. 10.3201/eid1303.06054917552092PMC2725917

[2] Rochelle PA, Marshall MM, Mead JR, Johnson AM, Korich DG, Rosen JS, Comparison of in vitro cell culture and mouse assay for measuring infectivity of *Cryptosporidium parvum.* Appl Environ Microbiol. 2002;68:3809–17. 10.1128/AEM.68.8.3809-3817.200212147476PMC124000
